# Physiological and transcriptomic analyses provide new insights into the effects of 1-MCP and ethephon treatments on apricot fruit during storage

**DOI:** 10.3389/fpls.2026.1744757

**Published:** 2026-02-17

**Authors:** Xinju Sun

**Affiliations:** Nanjing Normal University of Special Education, Nanjing, Jiangsu, China

**Keywords:** 1-MCP, apricot, ethephon, fruit softening, postharvest

## Abstract

Rapid ripening and a short postharvest shelf life are major issues limiting the apricot industry, and ethylene plays a significant role in these processes. In this study, ‘Fengyuanhong’ apricot fruits were treated with ethephon or 1-MCP and stored under controlled conditions. Physiological quality attributes (color, firmness, weight loss, soluble solids, sugars, and acids) were monitored over time. Multi time point transcriptome sequencing (RNA seq) was performed to compare gene expression profiles. Compared with the control treatment, ethephon treatment significantly altered the physiological characteristics of apricot fruits during storage, including reducing the storage period by 2 days, markedly changing the color parameters (L*, a*, b*, and h°), accelerating yellowing and water loss, and decreasing firmness. In contrast, compared with the control treatment, the 1-MCP treatment extended the storage period by 2 days, delayed fruit yellowing, and maintained firmness and stable water levels. Furthermore, no consistent patterns were observed in the levels of soluble solids, sugars, or acid-related indicators between the two treatments. Transcriptome analysis revealed that both treatments affected key metabolic pathways, including ribosome, energy metabolism, and signal transduction pathways. A co-expression network analysis revealed 16 ethylene pathway-related genes. This study demonstrated that 1-MCP treatment effectively extended both the storage duration and the shelf life of apricots. Furthermore, this study revealed the molecular mechanisms underlying the effects of both ethephon and 1-MCP treatments on postharvest apricot fruit, providing valuable scientific reference information for apricot preservation strategies.

## Highlights

- Ethephon accelerates while 1-MCP delays apricot fruit ripening during storage.
- Effects of ethylene/1-MCP treatment on the physiology and transcriptome of apricot fruits were elucidated.
- Sixteen potential regulatory genes were identified in postharvest apricot fruit.

## 1 Introduction

Apricot (*Prunus armeniaca* L.), a plant species belonging to the Rosaceae family, Amygdaloideae subfamily, and Prunus genus, is a fruit tree indigenous to China and ranks among the top three most widely cultivated stone fruits worldwide. It has remarkable stress resistance, broad adaptability, rich genetic diversity, versatile processing applications, and significant potential for value-added utilization. Moreover, apricot fruits have nutritional value and contain large amounts of β-carotene, essential vitamins, and various flavonoids, such as phenolic acids, flavonols, and flavanols, which contribute to their health-promoting properties (Ali et al., 2015).

However, as typical climacteric fruits, apricots exhibit intense postharvest respiration and excessive ethylene production, leading to rapid senescence, tissue softening, and decay. Their poor storability, limited transport tolerance, and short shelf life significantly decrease their commercial value. Consequently, elucidating the mechanisms underlying postharvest senescence in apricots and developing preservation technologies to regulate ripening processes, thereby inhibiting quality deterioration and extending storage duration, have emerged as crucial research focuses in postharvest fruit biology.

1-Methylcyclopropene (1-MCP), an ethylene receptor inhibitor, can effectively suppress the expression of genes involved in ethylene biosynthesis and consequently delay fruit ripening. Owing to its advantages, including stable chemical properties, nontoxicity, low effective dose and remarkable efficacy, 1-MCP has been widely applied to preserve various fruits and vegetables, such as apples (Cao et al., 2024), pears (Shu et al., 2020), peaches (Cai et al., 2018), bananas (Satekge and Magwaza, 2020) and durians (Thongkum et al., 2018). Ethephon, a synthetic compound widely used as a commercial ripening agent, effectively promotes endogenous ethylene biosynthesis during climacteric fruit ripening. As a gaseous plant hormone, ethylene regulates various aspects of plant growth and development. Moreover, ethylene is recognized as the key initiator of fruit ripening and senescence processes. It increases membrane permeability and disrupts cellular compartmentalization, ultimately accelerating fruit softening and senescence and reducing postharvest shelf life (Tipu and Sherif, 2024).

Currently, reports concerning the effects of individual 1-MCP or ethephon treatments and their combined applications with other regulators or hormones on apricot fruits are limited. For example (Li et al., 2002), reported that the delay in apricot ripening caused by 1-MCP was related to factors such as cultivar selection, fruit maturity, and application timing (Botondi et al., 2003). reported that 1-MCP treatment altered the composition of volatile compounds in apricot fruits, including a reduction in lactone synthesis and an increase in terpene alcohols. Studies by (Liang et al., 2023) and (Lv et al., 2023) revealed that compared with individual treatments alone, combined treatments of SA + 1-MCP and UV-C + 1-MCP, respectively, were more effective at preserving apricots.

Although the aforementioned studies have demonstrated the physiological effects of 1-MCP and ethephon on apricot fruit, the current understanding remains primarily at the level of phenotypic and basic biochemical changes. Systematic, genome-wide transcriptional analyses comparing the dynamic effects of ethylene promotion (via ethephon) and inhibition (via 1-MCP) on apricot fruit ripening and senescence are still lacking. Specifically, the molecular reprogramming triggered by these opposing treatments, the key genes and pathways governing ethylene-dependent quality deterioration, and the transcriptional basis for the extended shelf life conferred by 1-MCP in apricots remain largely unexplored. To address these gaps, this study employed a multi-timepoint transcriptome sequencing approach alongside detailed physiological profiling. We aimed to (1) systematically compare the effects of ethephon and 1-MCP on apricot fruit quality attributes; (2) delineate the dynamic transcriptional landscapes associated with accelerated versus delayed senescence; and (3) identify key candidate genes and regulatory networks underlying ethylene-mediated postharvest changes in apricots. Our findings provide novel molecular insights into the hormonal regulation of apricot fruit senescence and establish a valuable transcriptomic resource for future targeted breeding or biotechnological strategies aimed at improving postharvest longevity.

## 2 Materials and methods

### 2.1 Experimental materials and treatment

Uniform, undamaged ‘Fengyuanhong’ apricot fruits at the commercial ripening stage were collected and immediately transported to the laboratory. The fruits were subjected to the following treatments: A concentration of 1 μL·L_-1_ 1-MCP (Macklin Biochemical Technology Co., Ltd., Shanghai, China) was applied by airtight fumigation for 24 hours; a concentration of 30 mg·kg^−1^ ethephon (Macklin Biochemical Technology Co., Ltd.) was used to soak the apricot fruits for 20 min, followed by air drying and subsequent airtight storage for 24 hours; and a blank airtight treatment was used as the control. All the treated samples were placed in foam boxes and stored at 25 °C with 70% relative humidity. Sampling was conducted at 0, 2, 4, 6, 8, 10, and 12 days posttreatment. A destructive sampling approach was adopted, in which a distinct set of at least 20 fruits was collected for analysis at each time point. No fruit measured at a given time point was reused in subsequent sampling events. A portion of the fruits was used for relevant physiological measurements, while the remainder was rapidly dissected snap-frozen in liquid nitrogen, and stored at −80 °C for subsequent analysis. Three biological replicates were performed for each treatment group.

### 2.2 Measurement of color difference, fruit firmness, and fruit weight

The color difference values of the apricot fruits at different time points were measured using a CR-400 colorimeter (Konica Minolta, Japan) (three biological replicates, five fruits per replicate). CIELAB software was used to measure the L*, a*, b*, and h* values. The L* value represents lightness from black (L*=0) to white (L*=100), a* indicates the green (−) to red (+) spectrum, b* represents the blue (−) to yellow (+) spectrum, and h° denotes the hue angle, which ranges from 0° to 360° with specific color associations; different angles correspond to different colors. Usually, 0° (or 360°) is defined as red, 90° as yellow, 180° as green, and 270° as blue. Fruit firmness was measured using a fruit hardness tester (GY-2). The weight of individual apricot fruits from each treatment was determined using an electronic balance.

### 2.3 Determination of soluble solid, soluble sugar and organic acid contents

The soluble solids content was measured using a handheld refractometer (Atago, Japan, PAL-1). The concentrations of fructose, glucose, sorbitol, sucrose, malic acid, citric acid, quinic acid, shikimic acid, and oxalic acid were analyzed by high-performance liquid chromatography (HPLC; Thermo Scientific UltiMate 3000 system) following the chromatographic column specifications and detection methods described by Cheng et al. (2018), where 5.0 g of fruit flesh was weighed into a 5 mL grinding tube with zirconium oxide beads for homogenization, transferred to a 50 mL centrifuge tube and diluted to 10 mL with ultrapure water, subjected to an 80 °C water bath for 30 min with vortex mixing every 10 min, followed by 30 min of ultrasonic extraction (with one repetition of this water bath-ultrasonic cycle), then centrifuged at 12,000 rpm for 30 min at 4 °C, and finally filtered through a 0.22 μm aqueous membrane with the filtrate stored in amber vials for HPLC analysis (Cheng et al., 2018).

The sugar components were analyzed using a mobile phase consisting of acetonitrile (with 1% ammonia water) and water (85:15, v/v) at a flow rate of 0.2 mL/min, with the column temperature maintained at 45 °C, an injection volume of 2 μL, and a run time of 18 minutes, employing an ELSD detector with the nebulizer gas pressure set at 25 psi, drift tube temperature at 55 °C, and spray chamber temperature at 25 °C, using a UPLC ACQUITY BEH amide column (1.7 μm, 2.1×100 mm), whereas the organic acid components were analyzed with standard curves established from reference standards and their corresponding peak areas and 0.02 mol/L potassium hydrogen phosphate buffer (pH adjusted to 2.4 with phosphoric acid) as the mobile phase at a flow rate of 0.5 mL/min. The quantification of both the sugar and organic acid components was achieved by calculations against standard curves established from reference standards and their corresponding peak areas. The information of sampling time points for all experiments are provided in **Supplementary Table 1**.

### 2.4 RNA extraction, library preparation, and sequencing

Total RNA was extracted from frozen apricot fruit pulp following the protocol of the E.Z.N.A.^®^ Plant RNA Kit (Omega Bio-tek, USA). RNA integrity was assessed using a 2100 Bioanalyzer (Agilent Technologies, USA), and concentration and purity were measured using a NanoDrop 2000 spectrophotometer (Thermo Fisher Scientific, USA). RNA samples that satisfied the following quality criteria were classified as high-quality and used for subsequent library preparation: an OD260/280 ratio between 1.8 and 2.2, an OD260/230 ratio ≥ 2.0, an RNA integrity number (RIN) ≥ 6.5, a 28S:18S ribosomal RNA ratio ≥ 1.0, and a total RNA quantity exceeding 10 µg.

RNA-seq transcriptome libraries were constructed from 1 µg of total RNA per sample using a standard mRNA-seq workflow. Polyadenylated mRNA was enriched with oligo(dT) magnetic beads and then fragmented. First- and second-strand cDNA synthesis was performed, followed by end repair, adenylation of 3’ ends, and ligation of indexed adapters. The resulting cDNA libraries were size-selected (200–300 bp) on a 2% low range ultra agarose gel and amplified for 15 cycles with Phusion High-Fidelity DNA Polymerase (New England Biolabs, USA). Library concentration and size distribution were quantified with a TBS380 fluorometer (Turner BioSystems). Finally, paired-end sequencing (2 × 150 bp) was carried out on an Illumina NovaSeq 6000 platform (Illumina, USA) by Shanghai BIOZERON Biotech. Co., Ltd., following the manufacturer’s standard protocols.

### 2.5 Preprocessing, quality control, and sequence alignment of RNA sequencing data

Quality assessment and preprocessing were first performed on the raw sequencing data, where FastQC software was used to evaluate the quality metrics (including sequence quality distribution, GC content, and adapter contamination) of the original FASTQ files, after which Trimmomatic was used to remove low-quality sequences and adapter contaminants to obtain high-quality clean reads for downstream analysis. The preprocessed RNA-seq data were subsequently aligned to the ‘Jintaiyang’ reference genome (https://www.rosaceae.org/Analysis/10254125) using HISAT2 with the parameter ‘--dta’, after which the alignment results were sorted and indexed using SAMtools to generate BAM files for subsequent analyses (Jung et al., 2007).

### 2.6 Transcriptome assembly and quantification, differential gene expression analysis, and weighted gene co-expression network analysis

Transcriptome assembly and expression quantification were performed using StringTie2, followed by differentially expressed gene (DEG) screening and statistical analysis with the DESeq2 package in R, while the WGCNA package in R was employed to construct gene co-expression networks (criteria: average read count >10, fold change >2, adjusted p<0.05) with a soft threshold power β=12 to identify co-expression modules and analyze their phenotypic correlations. Finally, Cytoscape was used for network visualization of key modules to elucidate gene co-expression relationships and topological structures.

### 2.7 Identification of putative downstream target genes of transcription factors

Putative downstream target genes of the transcription factors (TFs) were identified using a co−expression network constructed with WGCNA (v1.71). Genes exhibiting a connection weight of ≥ 0.08 to each target TF were extracted as initial candidates. For each candidate gene, a 2 kb genomic region upstream of the translation start site was retrieved. These promoter sequences were scanned for the presence of known binding motifs of ERF and MYB transcription factors using the cis−regulatory element database integrated in plantTFDB (https://planttfdb.gao-lab.org/prediction.php). Candidate genes harboring the corresponding TF−binding motifs in their promoters were retained for further analysis. Finally, functional annotation of the filtered candidate target genes was performed using eggnog−mapper (emapper−2.1.13) with the eggNOG database (version 5.0.2).

### 2.8 Quantitative real−time PCR validation

To experimentally validate the transcriptome sequencing results, 8 key DEGs were selected for qRT–PCR analysis. The qRT-PCR method was performed with slight modifications based on the protocol previously described by Wu et al. (2017). First-strand cDNA was synthesized from 1 μg of total RNA using a PrimeScript RT reagent kit (Takara, China). qRT–PCR was conducted on a QuantStudio 5 real−time PCR system (Applied Biosystems, USA) in a 20 μL reaction mixture containing 10 μL SYBR Premix Ex Taq II (Takara), 0.8 μL each of forward and reverse primers (10 μM), 2 μL cDNA template, and 6.4 μL ddH_2_O. The amplification protocol consisted of initial denaturation at 95°C for 30 s, followed by 40 cycles of denaturation at 95°C for 5 s and annealing/extension at 60°C for 30 s. All reactions were carried out in triplicate. The actin gene of apricot was employed as the internal reference for normalization. Relative expression levels were calculated using the 2^−ΔΔCt^ method, and statistical significance between treatment and control groups was assessed by Student’s t−test (*P < 0.05). The sequences of the primers used are provided in **Supplementary Table 2**.

## 3 Results

### 3.1 Effects of 1-MCP and ethephon treatments on postharvest apricot fruit phenotypes

**Figure 1:**
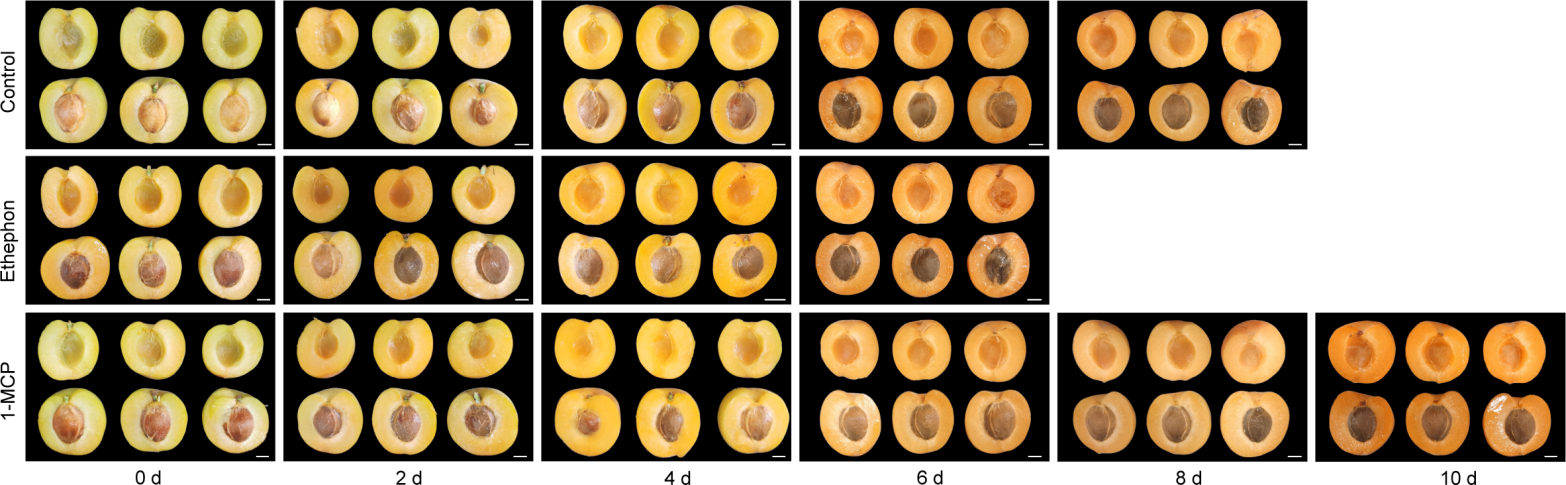
Phenotypic characteristics of apricot fruits over time following 1-MCP and ethephon treatments. The data for “Day 0” represent the initial state measured immediately after the completion of the respective treatments (1-MCP fumigation, ethephon immersion, or control treatment).

Treatment of mature ‘Fengyuanhong’ apricot fruits with 1-MCP or ethephon resulted in markedly divergent effects during 10 days of storage, with phenotypic observations performed every two days (**Figure 1**). The apricot fruits of the control group retained marketable quality—characterized by a bright orange–yellow color and the absence of decay—until day 8. While the visual appearance, particularly color development, of 1-MCP-treated fruits did not differ markedly from that of the control during the initial storage phase (e.g., through day 4), the treated fruits preserved these quality attributes until day 10, demonstrating a significant extension of shelf life. In contrast, ethephon treatment significantly accelerated the aging of apricot fruits. After the completion of all the treatments (i.e., day 0), the fruits in the ethephon group exhibited brown coloration, whereas those in both the control and 1-MCP groups retained their green color (**Figure 1**). The fruits treated with ethephon had completely rotted by day 8, and their color changed from green to yellow. With increasing storage time, the intensity of the yellow color increased (**Figure 1**).

### 3.2 Effects of 1-MCP and ethephon treatments on the color, firmness and per-fruit weight of postharvest apricot fruits

**Figure 2:**
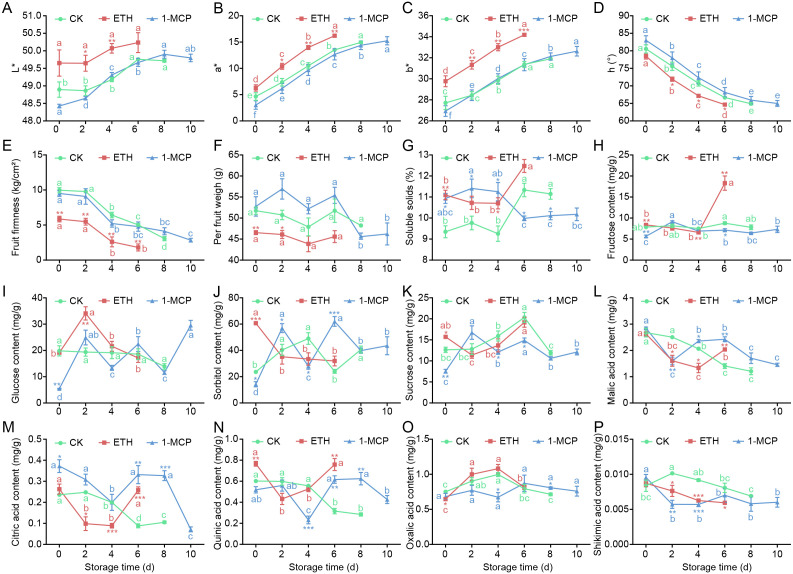
Physiological parameters of apricot fruits following ethephon and 1-MCP treatments. **(A)** L* value, **(B)** a* value, **(C)** b* value, and **(D)** h° measured by a colorimeter; **(E)** firmness; **(F)** per-fruit weight; **(G)** soluble solids content; sugar components, including **(H)** fructose, **(I)** glucose, **(J)** sorbitol, and **(K)** sucrose; organic acid components, including **(L)** malic acid, **(M)** citric acid, **(N)** quinic acid, **(O)** oxalic acid, and **(P)** shikimic acid. The data are presented as the means ± SEs (n=3). Asterisks indicate significant differences between the treatment group and the control at the same time point as determined by Student’s two-tailed *t* test (*P < 0.05, **P < 0.01, ***P < 0.001). Different lowercase letters denote significant differences among time points within the same treatment group at the 5% level based on ANOVA.

To evaluate the effects of 1-MCP and ethephon treatments on the physicochemical characteristics of apricot fruits, we first measured the surface color parameters using a colorimeter (**Figures 2A–D**). The results demonstrated that while 1-MCP treatment resulted in no significant differences in color indices compared with those of the control, ethephon treatment markedly altered fruit coloration during postharvest storage (**Figures 2A–D**). Specifically, ethephon treatment increased the L*, a*, and b* values by 0.96%–1.81% (*P* = 0.0039–0.1682), 19.85%–41.16% (*P* = 0.0031–0.0288), and 8.97%–10.52% (*P* = 0.0008–0.0056), respectively, but significantly decreased the h° value by 3.04%–5.02% during postharvest days 2–6 (**Figures 2A–D**). Further analysis of fruit firmness and fruit weight during storage (**Figures 2E, F**) revealed no significant differences in firmness between the 1-MCP-treated group and the control group, whereas compared with the control treatment, the ethephon treatment resulted in a significant reduction in firmness of 41.22%–63.97% (*P* = 0.0011–0.0092) during postharvest storage (**Figure 2E**). Additionally, compared with control fruits, ethephon-treated fruits presented significant decreases in per-fruit weight of 10.07% (*P* = 0.0060) and 9.16% (*P* = 0.0347) on storage days 0 and 2, respectively (**Figure 2F**).

### 3.3 Effects of 1-MCP and ethephon treatments on the flavor profiles of postharvest apricot fruits

To investigate the effects of 1-MCP and ethephon treatments on apricot fruit flavor quality, we analyzed the contents of soluble solids, sugars, and organic acids. The results demonstrated that both treatments significantly altered the soluble solids content during postharvest storage. Ethephon treatments increased the soluble solids content by 18.76% (*P* = 0.0082) and 15.59% (*P* = 0.0328), respectively, 0 and 4 days after treatment. The 1-MCP treatment increased the soluble solids content by 16.83%–21.57% (*P* = 0.0123–0.0396) 0–4 days after treatment, whereas the 1-MCP treatment decreased the soluble solids content by 11.88% (*P* = 0.0160) and 9.27% (*P* = 0.0314), respectively, 6 and 8 days after treatment (**Figure 2G**).

Significant variations were observed in the sugar and organic acid profiles among the treatments. Compared with the control treatment, the ethephon treatment increased the fructose content by 6.26% (*P* = 0.0023) and 108.33% (*P* = 0.0053) 0 and 6 days after treatment, respectively, but decreased it by 12.04% (*P* = 0.0093) 4 days after treatment, whereas the 1-MCP treatment reduced the fructose content by 27.88% (*P* = 0.0016) and 18.80% (*P* = 0.0219) 0 and 6 days after treatment, respectively (**Figure 2H**). Ethephon treatment increased the glucose content by 76.02% (*P* = 0.0076) 2 days after treatment, whereas 1-MCP treatment decreased it by 72.57% (*P* = 0.0018) 0 d after treatment (**Figure 2I**). With respect to sorbitol, 1-MCP treatment resulted in dynamic changes, with 39.71% (*P* = 0.0167) and 42.96% (*P* = 0.0129) decreases 0 and 4 days after treatment, respectively, but 41.01% (*P* = 0.0380) and 157.97% (*P* = 0.0007) increases 2 and 6 days after treatment, respectively, whereas ethephon treatment resulted in a 156.93% (*P* = 0.00002) increase 0 days after treatment (**Figure 2J**). The sucrose content decreased by 40.36% (*P* = 0.0027), 23.99% (*P* = 0.0429), and 26.89% (*P* = 0.0142) 0, 4, and 6 days after treatment with 1-MCP, respectively, whereas ethephon treatment increased it by 24.41% (*P* = 0.0104) 0 days after treatment (**Figure 2K**).

In terms of organic acids, ethephon treatment consistently reduced the malic and citric acid contents 2 and 4 days after treatment but they increased after 6 days (**Figures 2L, M**). 1-MCP treatment decreased the malic acid content by 36.12% (*P* = 0.0040) after 2 days but it increased by 14.08% (*P* = 0.0105) and 72.33% (*P* = 0.0015) after 4 and 6 days, respectively, whereas it dramatically increased the citric acid content by 276.34% (*P* = 0.0051) and 210.67% (*P* = 0.0008) after 6 and 8 days, respectively (**Figures 2L, M**). Quinic acid increased by 27.36% (*P* = 0.0040) and 140.01% (*P* = 0.0021) 0 and 6 days after ethephon treatment, respectively, whereas 1-MCP treatment caused a 58.29% (*P* = 0.0009) decrease 4 days after treatment, followed by 94.88% (*P* = 0.0025) and 119.30% (*P* = 0.0051) increases after 6 and 8 days, respectively (**Figure 2N**). The oxalic acid content decreased by 13.84% (*P* = 0.0343) after ethephon treatment (0 days), whereas 1-MCP treatment resulted in a 32.45% (*P* = 0.0109) decrease and a 13.22% (*P* = 0.0160) increase after 4 and 8 days, respectively (**Figure 2O**). The shikimic acid content decreased by 24.75%–31.85% (*P* = 0.0004–0.0375) in ethephon-treated fruits from 2–6 days, whereas 1-MCP treatment resulted in greater reductions of 43.69% (*P* = 0.0020) and 37.79% (*P* = 0.0007) after 2 and 4 days, respectively (**Figure 2P**).

### 3.4 Transcriptome analysis

#### 3.4.1 RNA-seq data quality assessment

To elucidate the molecular basis underlying the effects of ethephon and 1-MCP treatments on the physiological and biochemical indices of apricot fruit during storage, we conducted transcriptome sequencing analysis on 11 sample groups (the control group and 1-MCP-treated groups at 2 d, 4 d, 6 d, and 8 d of storage and the ethephon-treated groups at 2 d, 4 d, and 6 d of storage). After quality control and data filtering, a total of 9,023,158,900 average clean bases and 60,154,392.67 average clean reads were obtained. The clean reads were then aligned to the ‘Jintaiyang’ reference genome. The average number of reads aligned, percentage of reads aligned, number of unique mapping reads, percentage of unique mapping reads, and Q30 were 49944547.42, 82.62%, 47191301.3, 78.14% and 83.59%, respectively (**Supplementary Table 3**). These results indicate that the RNA-seq data are of high quality and can be used for further analysis.

**Figure 3:**
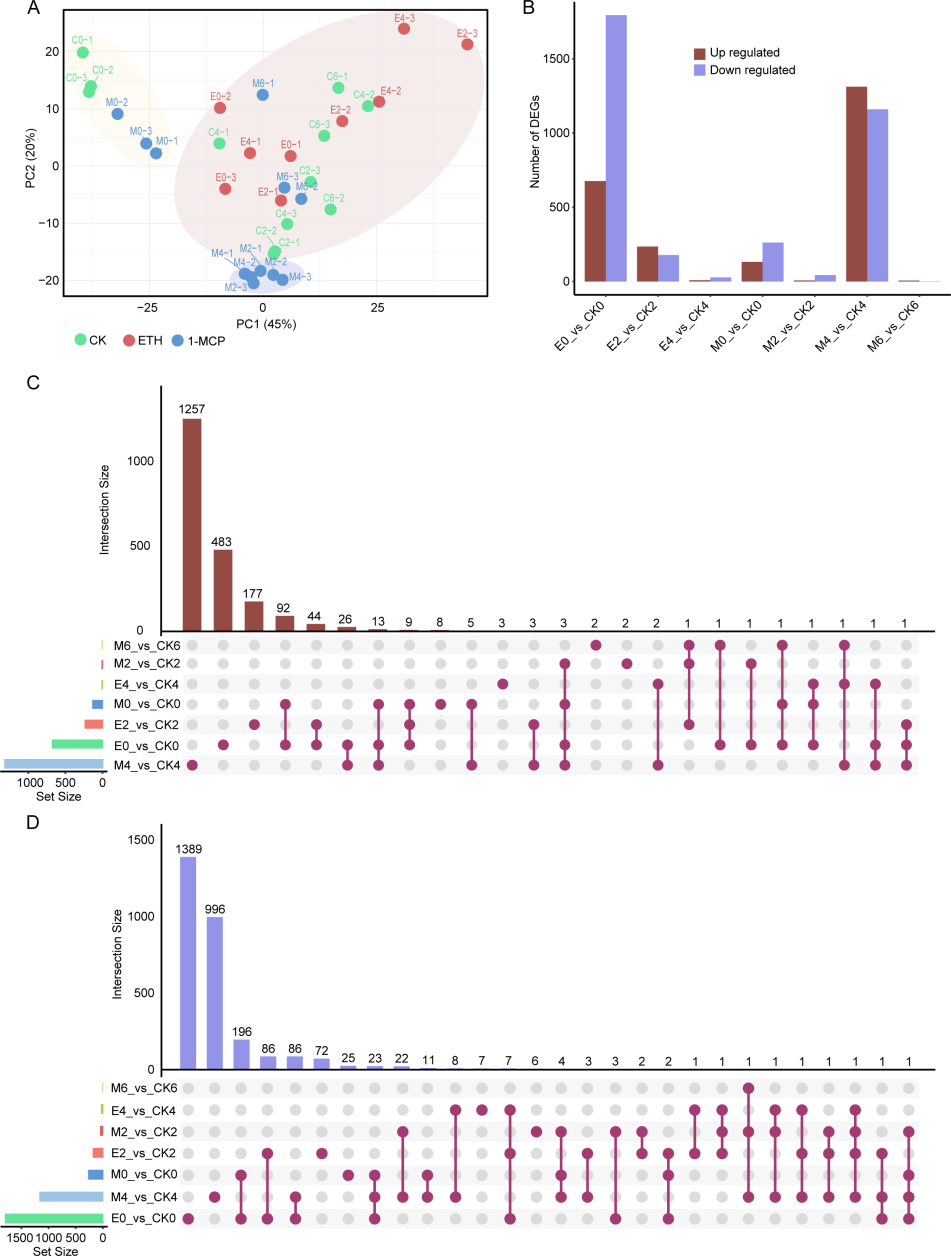
Transcriptomic PCA and DEG analysis of apricot fruits treated with ethephon and 1-MCP. **(A)** PCA of the transcriptome data. The suffixes “_1”, “_2”, and “_3” following the sample IDs represent the three biological replicates. **(B)** Bar plot showing statistics of upregulated and downregulated DEGs among different comparison groups. **(C)** UpSet plot displaying overlapping upregulated DEGs among different comparison groups. **(D)** UpSet plot illustrating overlapping downregulated DEGs among the comparison groups. The number of DEGs on the left **(C, D)** is consistent with that in **(B)**. The matrix of dots below illustrates the intersection relationships. C0, C2, C4, and C6 represent the control group samples at 2, 4, 6, and 8 days of storage, respectively; E0, E2, and E4 represent the ethephon-treated group samples at 2, 4, and 6 days of storage, respectively; and M0, M2, M4, and M6 represent the 1-MCP-treated group samples at 2, 4, 6, and 8 days of storage, respectively.

On the basis of the expression levels of 32,636 genes identified from the transcriptome data, we performed a principal component analysis (PCA) to assess the differences in transcriptomic profiles among the samples. PC1 accounted for 45% of the variance, whereas PC2 explained 20%. All the samples could be grouped into three main clusters on the basis of their transcriptomic profiles (**Figure 3A**). Cluster I consisted of control samples and 1−MCP−treated samples at 2 days of storage, which clustered closely together, indicating that 1−MCP had a relatively limited overall impact on the apricot fruit transcriptome during early storage. Cluster II comprised 1−MCP−treated samples at 4 and 6 days of storage, demonstrating that 1−MCP induced a distinct and stable shift in gene expression patterns during mid−storage. Cluster III included all remaining samples (control and 1−MCP−treated samples at other time points, as well as all ethephon−treated samples) and exhibited a mixed distribution, suggesting a tendency for ethephon−treated samples to aggregate with later−stage samples from other groups. This pattern aligns with the physiological effect of ethephon treatment, which significantly accelerates fruit senescence and leads to rapid convergence of transcriptomic states (**Figure 3A**).

#### 3.4.2 DEG analysis

We further analyzed the differential expression patterns of genes in apricot fruits subjected to different treatments. The results revealed that the total numbers of DEGs in the comparisons E0_vs_CK0, E2_vs_CK2, E4_vs_CK4, M0_vs_CK0, M2_vs_CK2, M4_vs_CK4, and M6_vs_CK6 were 2,470 (676 upregulated; 1,794 downregulated), 413 (235 upregulated; 178 downregulated), 35 (8 upregulated; 27 downregulated), 394 (132 upregulated; 262 downregulated), 50 (7 upregulated; 43 downregulated), 2,472 (1,312 upregulated; 1,160 downregulated), and 7 (6 upregulated; 1 downregulated), respectively (**Figure 3B**). Among the four comparison groups (M2_vs_CK2, M0_vs_CK0, E0_vs_CK0, and M4_vs_CK4), three overlapping upregulated DEGs were identified (**Figure 3C**). Additionally, in two sets of four comparison pairs (E4_vs_CK4, M2_vs_CK2, E2_vs_CK2, and M4_vs_CK4; M2_vs_CK2, M0_vs_CK2, M4_vs_CK4, and E0_vs_CK0), one overlapping downregulated DEG was identified in each set (**Figure 3D**). These genes may play potential housekeeping roles in apricot fruits during storage after ethephon and 1-MCP treatments. Furthermore, unique DEGs with distinct expression changes were detected in the following samples: M6_vs_CK6 (2 DEGs, both upregulated), M2_vs_CK2 (8 DEGs: 2 upregulated, 6 downregulated), E4_vs_CK4 (10 DEGs: 3 upregulated, 7 downregulated), M0_vs_CK0 (33 DEGs: 8 upregulated, 25 downregulated), E2_vs_CK2 (249 DEGs: 177 upregulated, 72 downregulated), E0_vs_CK0 (1,872 DEGs: 483 upregulated, 1,389 downregulated), and M4_vs_CK4 (2,253 DEGs: 1,257 upregulated, 996 downregulated) (**Figures 3C, D**).

Moreover, we identified 339 TFs from 42 TF families whose expression significantly differed (**Supplementary Figure 1A**). Compared with the control, both the ethephon treatment and the 1-MCP treatment resulted in significantly more downregulated than upregulated TFs in apricot fruits at all time points except for the 1-MCP treatment 8 days after treatment. This pattern suggests that these treatments primarily induce transcriptional repression, whereas 1-MCP treatment may trigger late-stage transcriptional activation (**Supplementary Figure 1B**). Among the three comparison groups (E2_vs_CK2, M4_vs_CK4, and E0_vs_CK0), two overlapping differentially expressed TFs (DETFs) were identified (**Supplementary Figure 1C**). Additionally, in four sets of three comparison pairs (E4_vs_CK4, E2_vs_CK2, and E0_vs_CK0; M6_vs_CK6, M0_vs_CK0, and E0_vs_CK0; M0_vs_CK0, M4_vs_CK4, and E0_vs_CK0), one overlapping DETF was identified in each set (**Supplementary Figure 1C**). Notably, unique DETF patterns were identified in specific comparisons: 120 DETFs were identified in E0_vs_CK0, 94 DETFs were identified in M4_vs_CK4, 10 DETFs were identified in E2_vs_CK2, and 3 DETFs were identified in M0_vs_CK0 (**Supplementary Figure 1C**).

#### 3.4.3 Functional analysis of DEGs

**Figure 4:**
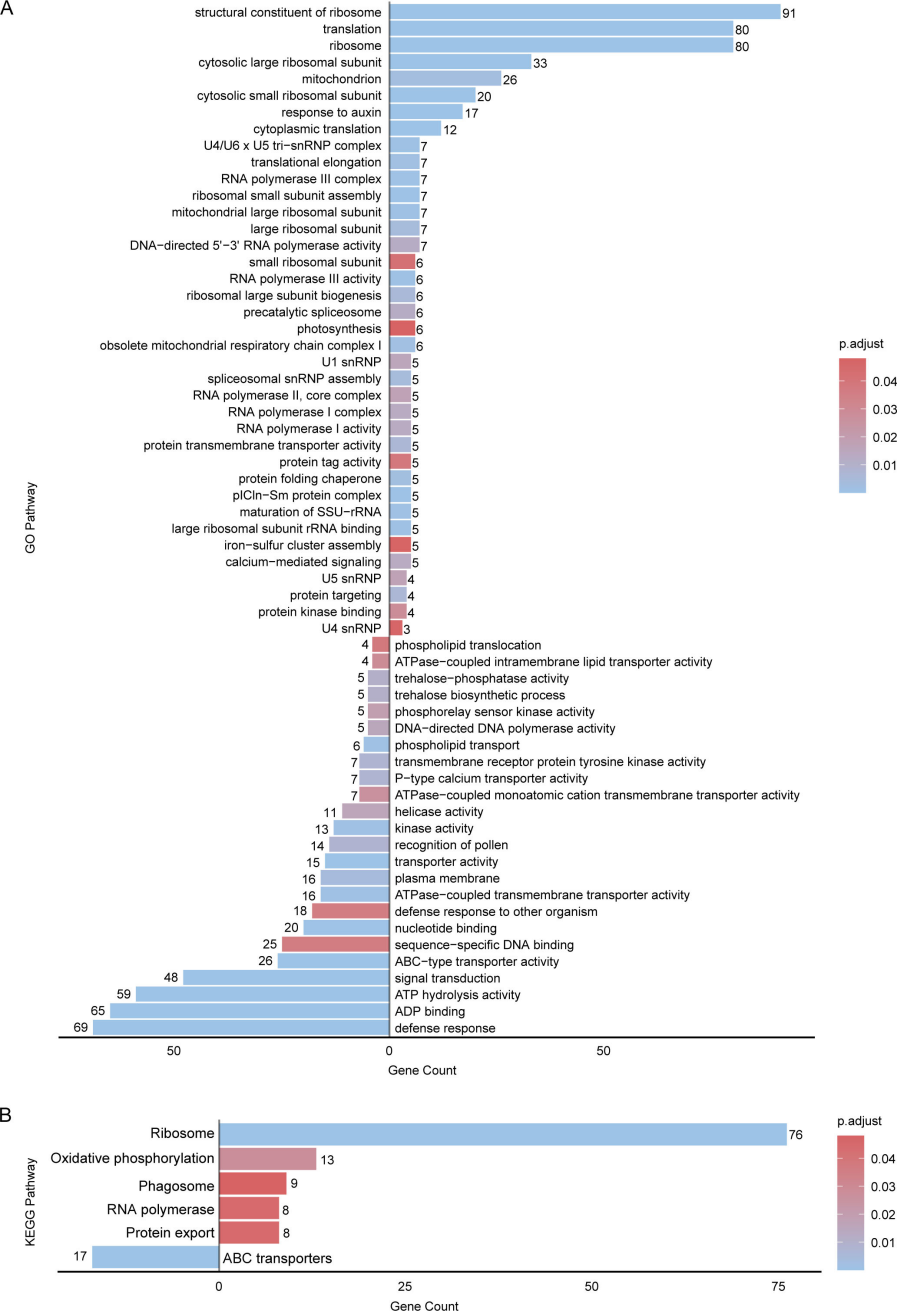
GO **(A)** and KEGG **(B)** enrichment analyses of DEGs in apricot fruits treated with ethephon and 1-MCP. The bar plot displays the enrichment results for downregulated DEGs (left) and upregulated DEGs (right). The color gradient of the bars corresponds to the range of p−values, with darker/lighter hues indicating lower/higher statistical significance.

To elucidate the biological functions of the identified DEGs, we performed Gene Ontology (GO) and Kyoto Encyclopedia of Genes and Genomes (KEGG) enrichment analyses. GO analysis revealed that 38 GO terms were significantly enriched in the upregulated genes, primarily structural constituents of ribosomes (91 genes), translation (80 genes), and ribosomes (80 genes), whereas 24 GO terms were associated with the downregulated genes, such as defense response (69 genes), ADP binding (65 genes), ATP hydrolysis activity (59 genes), and signal transduction (48 genes) (**Figure 4A**). KEGG pathway analysis revealed that the upregulated genes were associated with 5 major pathways: ribosome (76 genes), oxidative phosphorylation (13 genes), phagosome (9 genes), RNA polymerase (8 genes), and protein export (8 genes). In addition, 17 downregulated genes were associated with a single pathway, the ABC transporter pathway (**Figure 4B**).

#### 3.4.4 WGCNA

**Figure 5:**
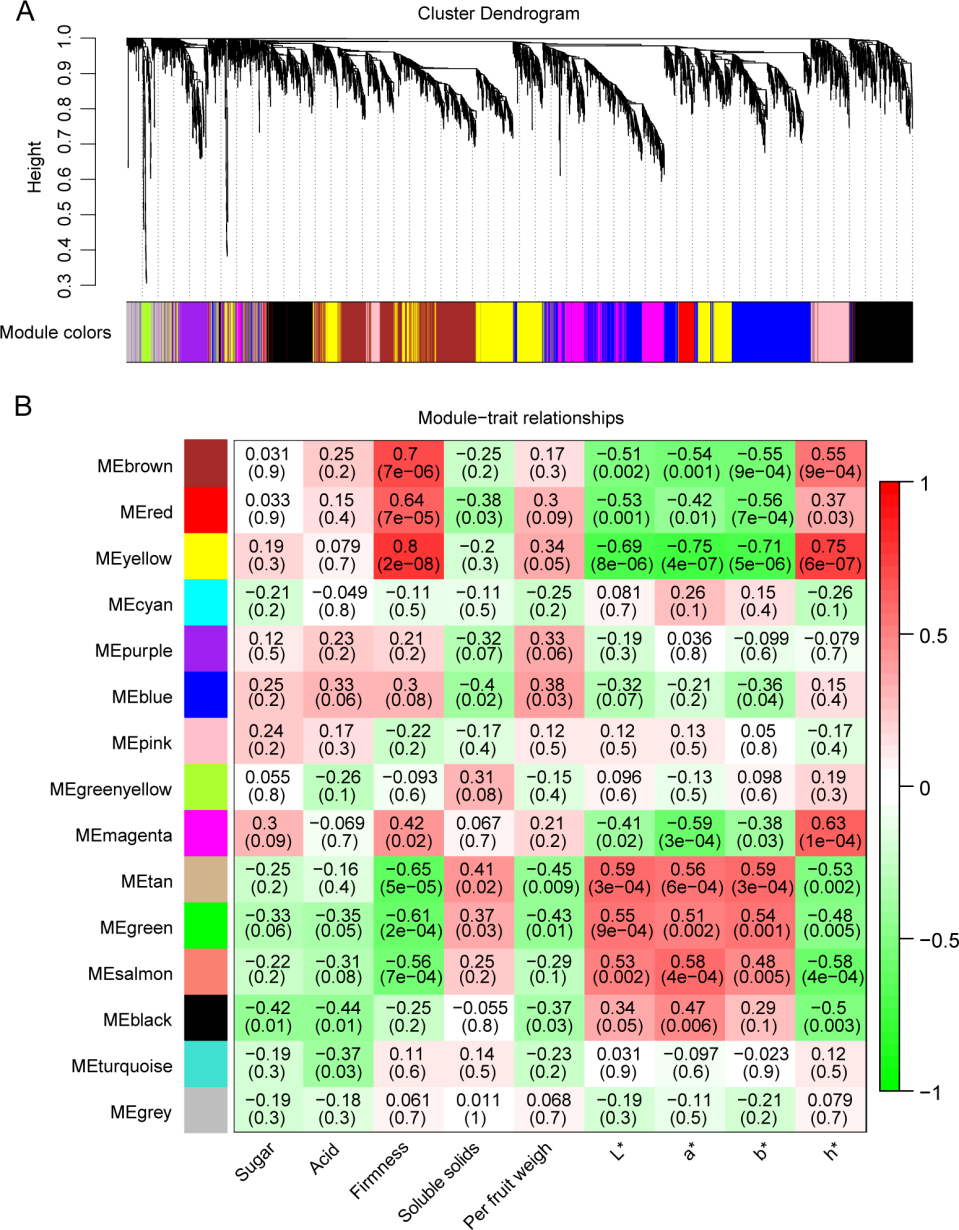
WGCNA of apricot fruits treated with ethephon and 1-MCP. **(A)** Hierarchical gene clustering dendrogram and identification of co-expression modules. **(B)** Heatmap displaying correlations between all modules and physiological quality indices. The left color bar indicates distinct modules; numerical values in grids represent Pearson correlation coefficients and their significance levels between modules and quality traits; in the central heatmap, darker hues indicate stronger correlations (red: positive correlation; green: negative correlation).

**Figure 6:**
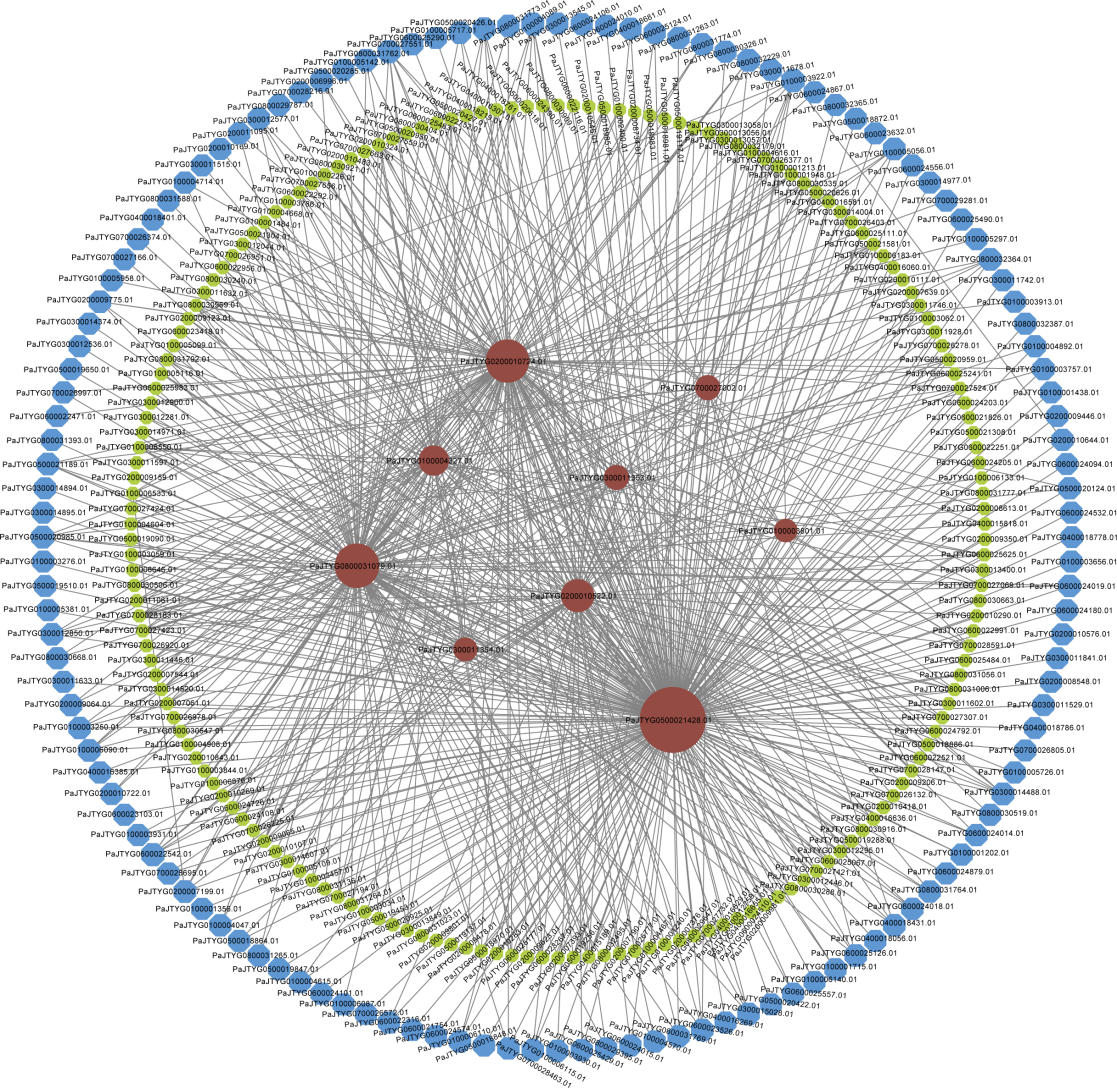
Gene regulatory network of the brown module identified by WGCNA. The blue blocks represent structural genes, the green blocks denote TFs, and the red blocks indicate ethylene pathway-related genes.

**Figure 7:**
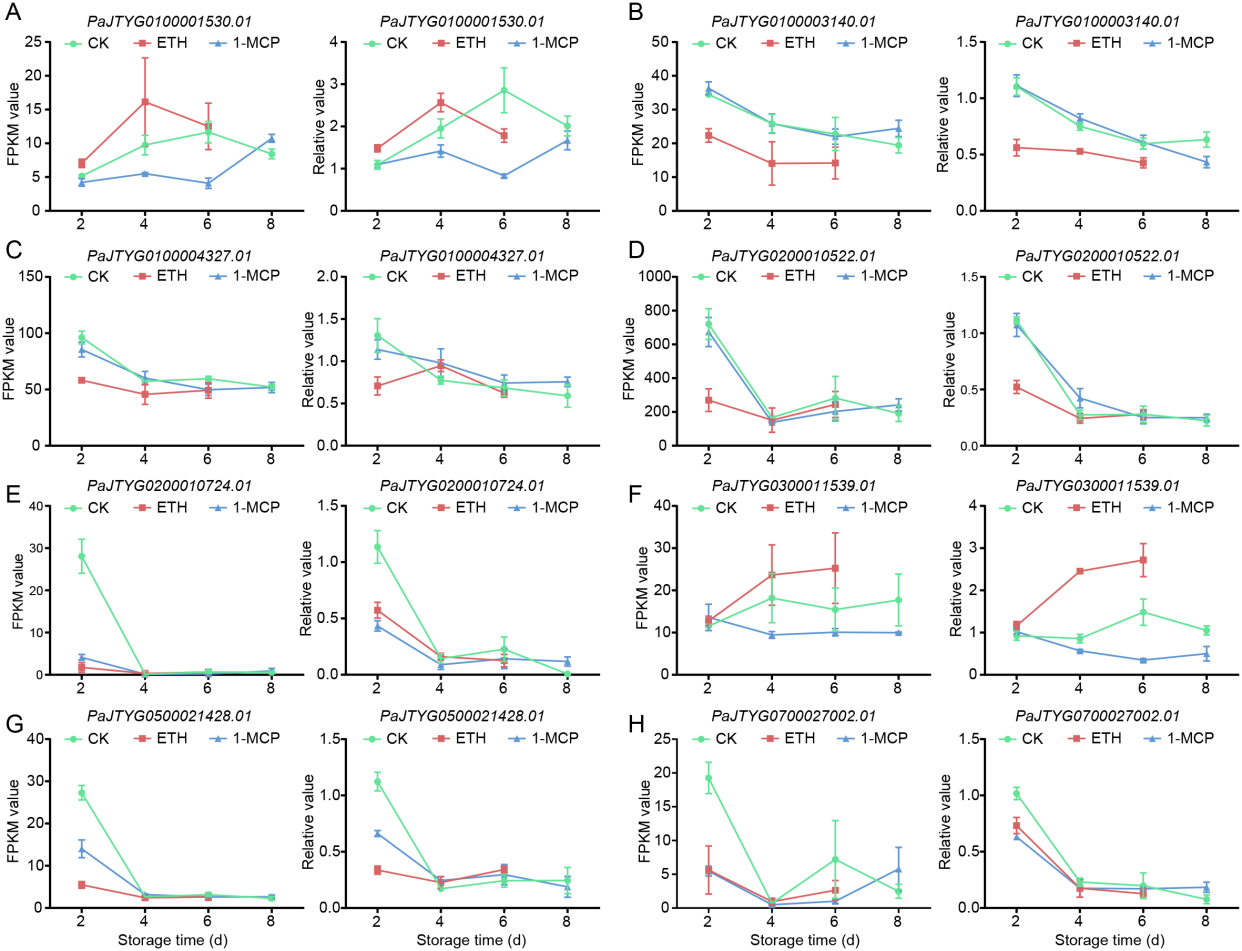
The expression patterns of 8 key ethylene pathway genes, selected based on WGCNA, were verified via qRT–PCR. **(A–D)** The left panel shows line charts of FPKM values derived from RNA-seq data for selected genes, while the right panel displays line charts of relative gene expression levels validated by qRT−PCR. The data are presented as the means ± SEs (n=3). **(A–H)** The left panel shows line charts of FPKM values derived from RNA-seq data for selected genes, while the right panel displays line charts of relative gene expression levels validated by qRT PCR.

To investigate the molecular mechanisms by which 1-MCP and ethephon treatments affect the physiological parameters of apricot fruit during storage, we conducted a WGCNA on 8,159 genes related to the sugar content, acid content, firmness, soluble solids content, per-fruit weight, and color indices (L*, a*, b*, h°) (**Figure 5**). The analysis revealed 15 co-expression modules, with the brown module containing the most genes (n=585) and the salmon module containing the fewest genes (n=9) (**Figure 5B**). Seven modules (brown, red, yellow, magenta, tan, green, and salmon) presented the strongest correlations with physiological indices, particularly those related to firmness and coloration, indicating that the 1-MCP and ethephon treatments primarily influence these quality attributes during storage (**Figure 5B**). Gene regulatory network analysis of these seven modules revealed that the brown module included 356 structural genes, 483 TFs, and 9 ethylene pathway-related genes (7 APETALA2/ethylene response factor (AP2/ERF) family members and 2 lysine histidine transporter (LHT) genes) (**Figure 6**); the red module included 88 structural genes, 112 TFs, and 2 ethylene pathway-related genes (1 *MYB1R1* and 1 *AP2/ERF*) (**Supplementary Figure 2**); the yellow module included 73 structural genes, 107 TFs, and 2 ethylene pathway-related genes (both *AP2/ERF*) (**Supplementary Figure 3**); the magenta module included 9 structural genes and 32 TFs (**Supplementary Figure 4**); the tan module included 11 structural genes and 21 TFs (**Supplementary Figure 5**); and the green module included 184 structural genes, 205 TFs, and 3 ethylene pathway-related genes (2 *AP2/ERF* and 1 1-aminocyclopropane-1-carboxylate oxidase (*ACO*)) (**Supplementary Figure 6**); and the salmon module included 12 structural genes and 9 TFs (**Supplementary Figure 7**). Given the close association between 1-MCP/ethephon treatments and ethylene signaling, the 16 identified ethylene pathway genes (12 AP2/ERF members and 1 *MYB1R1*, 1 *ACO*, and 2 *LHT* genes) likely represent key regulators mediating treatment-induced modifications of storage-related quality parameters in apricot fruit. We selected eight of these genes for qRT–PCR validation. The results showed that the FPKM values derived from RNA-seq data exhibited consistent trends with the qRT–PCR results, indicating high reliability of the transcriptomic data in this study (**Figure 7**).

To elucidate the downstream regulatory networks of the 13 ethylene−pathway−associated transcription factors (TFs), we performed a systematic prediction of their potential target genes. We first extracted gene sets directly linked to each target TF based on WGCNA. The results indicated that TF *PaJTYG0500021428.01* had the largest number of predicted downstream targets (261 genes), followed by *PaJTYG0800031079.01* (134 genes) and *PaJTYG0200010724.01* (127 genes), while *PaJTYG0100003140.01* showed the fewest potential targets (only 1 gene) (**Supplementary Figure 8A**). Co−expression edge weights between TFs and their putative targets were predominantly clustered around two values: 0.22 and 0.35 (**Supplementary Figure 8B**). Furthermore, to strengthen the reliability of these regulatory relationships, we scanned the upstream promoter regions of the candidate target genes and identified conserved binding motifs characteristic of the corresponding transcription factor families. Notably, for four of the 13 TFs (*PaJTYG0300011543.01*, *PaJTYG0300011541.01*, *PaJTYG0100001530.01*, and *PaJTYG0300011539.01*), no typical TF−binding motifs were detected in the promoters of their associated candidate targets (**Supplementary Figure 8C**). Finally, by integrating co−expression network weights and promoter cis−element features, we constructed a preliminary downstream target gene map for key ethylene−pathway TFs, which includes 315 target genes (**Supplementary Figure 8**; **Supplementary Table 4**). This work provides important clues for subsequent experimental validation and functional characterization.

## 4 Discussion

This study revealed that ethephon significantly accelerated apricot fruit ripening during storage, whereas 1-MCP markedly inhibited this progression, which is consistent with findings reported in apple (Yang et al., 2013), kiwifruit (Choi et al., 2023), pineapple and other species (Shiomi et al., 2002). The consumer preference of apricots is predominantly determined by fruit quality attributes, including color, firmness, soluble solids content, and sugar and acid levels. Previous studies have shown that 1-MCP treatment notably affects fruit coloration during storage. For example, it delays peel yellowing in bananas (Chang and Brecht, 2023), reduces yellowing in durian pulp (Thongkum et al., 2018), inhibits degreening in avocados (Jeong et al., 2002) and citrus (Porat et al., 1999), retards color deepening in medlars (*Mespilus germanica*) (Selcuk and Erkan, 2015) and strawberries (Tian et al., 2000), suppresses reddening in plum flesh (Pan et al., 2016), prevents browning in grape rachises (Li et al., 2015) and litchi pericarps (Sivakumar and Korsten, 2010), and paradoxically enhances red coloration in peaches (Zhang et al., 2022). This study revealed that ethephon treatment significantly accelerated apricot fruit yellowing by modifying colorimetric parameters (L*, a*, b* and h°), whereas 1-MCP treatment effectively inhibited this chromatic transition and extended the postharvest storage life of apricot fruits by 2 days, corroborating previous research findings (Fan et al., 2018). Notably, 1-MCP treatment demonstrates remarkable efficacy in retarding both fruit softening and moisture loss during storage, as exemplified by its ability to significantly mitigate firmness reduction and weight loss in multiple fruit species, including medlar (*Mespilus germanica*) (Selcuk and Erkan, 2015), kiwiberry (*Actinidia arguta*) (Xiong et al., 2023), avocado (*Persea americana*) (Jeong et al., 2002), plum (*Prunus domestica*) (Pan et al., 2016), and apple (*Malus domestica*) (Cao et al., 2024). Conversely, the observed acceleration of fruit softening and mass reduction by ethephon treatment was consistent with established physiological responses to ethylene stimulation.

Extensive research has shown that fruit flavor, particularly sugar and acid composition, often displays complex and variable responses to postharvest treatments (Wu et al., 2025; Xiong et al., 2023; Wang et al., 2018; Selcuk and Erkan, 2015). This complexity was also observed in the present study, where no consistent trend in sugar and acid profiles was detected. This variability likely originates from the dynamic balance inherent to sugar and acid metabolism, which integrates synthesis, degradation, interconversion, and transport processes. As a master ripening signal, ethylene simultaneously activates multiple metabolic cascades. Exogenous application of ethephon or 1-MCP disrupts this endogenous steady state, leading to temporally and quantitatively divergent responses across individual pathways. Moreover, such variability is strongly influenced by the specific developmental stage and tissue−specific microenvironment of the fruit. Our sampling strategy involved critical transitions from early maturity to senescence, during which time the effects of ethylene signaling dynamically shifted in accordance with the progression of the fruit’s physiological status. This plasticity suggests that, unlike more terminal traits such as color and softening—which are more directly and consistently modulated by ethylene—sugar and acid metabolism represents a more fundamental physiological layer whose regulation is highly plastic and likely involves substantial crosstalk with non−ethylene pathways.

The APETALA2/ethylene response factor (AP2/ERF) family encompasses crucial TFs in the ethylene signaling pathway that play pivotal roles in regulating fruit ripening and senescence processes, particularly in modulating pigment accumulation, fruit softening, aroma formation, and flavor modification (Gao et al., 2020; Zhai et al., 2022). As the key enzyme in ethylene biosynthesis, 1-aminocyclopropane-1-carboxylate oxidase (ACO) catalyzes the final step of the conversion of 1-aminocyclopropane-1-carboxylic acid (ACC) to ethylene. Studies in (*Solanum lycopersicum*) (Zhang et al., 2009) and banana (*Musa acuminata*) (Xiao et al., 2013) have shown that members of the ERF TF family can bind to promoter elements of *ACO* gene family members, thereby modulating ethylene signal transduction and physiological responses. *LHT1* is involved in the uptake of 1-aminocyclopropane-1-carboxylic acid (ACC) in *Arabidopsis*, and *LHT1* mutants also exhibit an early aging phenotype (Shin et al., 2015). Other members of the LHT family have similar expression patterns to *LHT1* and may also have ACC transport functions (Wu et al., 2023). Postharvest ethylene treatment upregulates the *MYB1R1* TF in kiwifruit (*Actinidia chinensis*) (Tilahun et al., 2020). Our current study, which involved integrated physiological and transcriptomic analyses, revealed 12 AP2/ERF family members, 1 *MYB1R1* gene, 1 *ACO* gene, and 2 *LHT* genes as putative key regulators of apricot fruit ripening and senescence in response to 1-MCP and ethephon treatments, potentially modulating ethylene biosynthesis pathways during storage (**Figure 6**, **Supplementary Figures 2–7**). The downstream 315 target genes of 13 key ethylene pathway transcription factors were further predicted (**Supplementary Figure 8**, **Supplementary Table 4**). We propose that these transcription factors may function cooperatively to regulate these targets, thereby collectively fine−tuning multiple key physiological processes during postharvest storage of apricot fruit. These target genes are functionally enriched in pathways related to cell wall remodeling and degradation (e.g., pectin methylesterase), stress and immune responses (e.g., late embryogenesis abundant protein, TMV resistance protein, disease resistance NB-LRR family proteins, disease resistance protein RPP8-like, and leucine-rich repeat extensin-like proteins), sugar and acid biosynthesis and metabolism (e.g., glycosyltransferase), secondary metabolism and pigment conversion (e.g., UDP-glucuronate 4-epimerase, glycosyltransferase, zeaxanthin epoxidase), senescence regulation (e.g., senescence-associated proteins), and intracellular signal transduction (e.g., calcium-binding proteins, calmodulin-like proteins) (**Supplementary Table 4**). These functional associations align closely with the observed phenotypic changes, including fruit softening, color transition, dynamic shifts in sugar and acid content, and senescence progression. Notably, the downstream target set also contains multiple ethylene−pathway−associated transcription factors, suggesting the potential amplification of ethylene signaling through transcriptional cascades that may further modulate these phenotypic traits.

## 5 Conclusion

Regulating ethylene levels is a common practice for supporting postharvest fruit quality. Our study demonstrated that ethephon significantly accelerated the ripening of apricot fruits during storage, specifically by reducing the storage duration by 2 days, promoting faster fruit yellowing, accelerating weight loss, and significantly reducing firmness. In contrast, 1-MCP effectively inhibited this ripening process and extended the storage period by 2 days. Further RNA-seq analysis revealed the molecular mechanisms underlying the effects of ethephon and 1-MCP treatments on physiological indicators related to apricot fruit storage. Additionally, WGCNA revealed 16 ethylene pathway-related genes. By combining co−expression network edge weights with promoter cis−regulatory element analysis, we systematically predicted downstream target genes for 13 of the corresponding transcription factors, resulting in a total of 315 candidate targets. This study highlights the significant potential of 1-MCP in postharvest apricot storage and provides candidate genetic resources for regulating ethylene levels in postharvest apricot fruits.

## Data Availability

All the raw transcriptome sequencing data have been deposited in the China National GeneBank Sequence Archive (CNSA) database (Guo et al., 2020) with accession number CNP0008354 https://db.cngb.org/data_resources/?query=CNP0008354.
