# Supplementary material for: Physiological and transcriptomic analyses provide new insights into the effects of 1-MCP and ethephon treatments on apricot fruit during storage
Source: Front Plant Sci. 2026 Feb 17;17:1744757. doi: 10.3389/fpls.2026.1744757 (PMC12953563; doi:10.3389/fpls.2026.1744757)
Supplement: Supplementary file 1 [file DataSheet1.docx]

**Supplementary Materials**

**Figures S1-S8**

**Tables S1-S4**

**
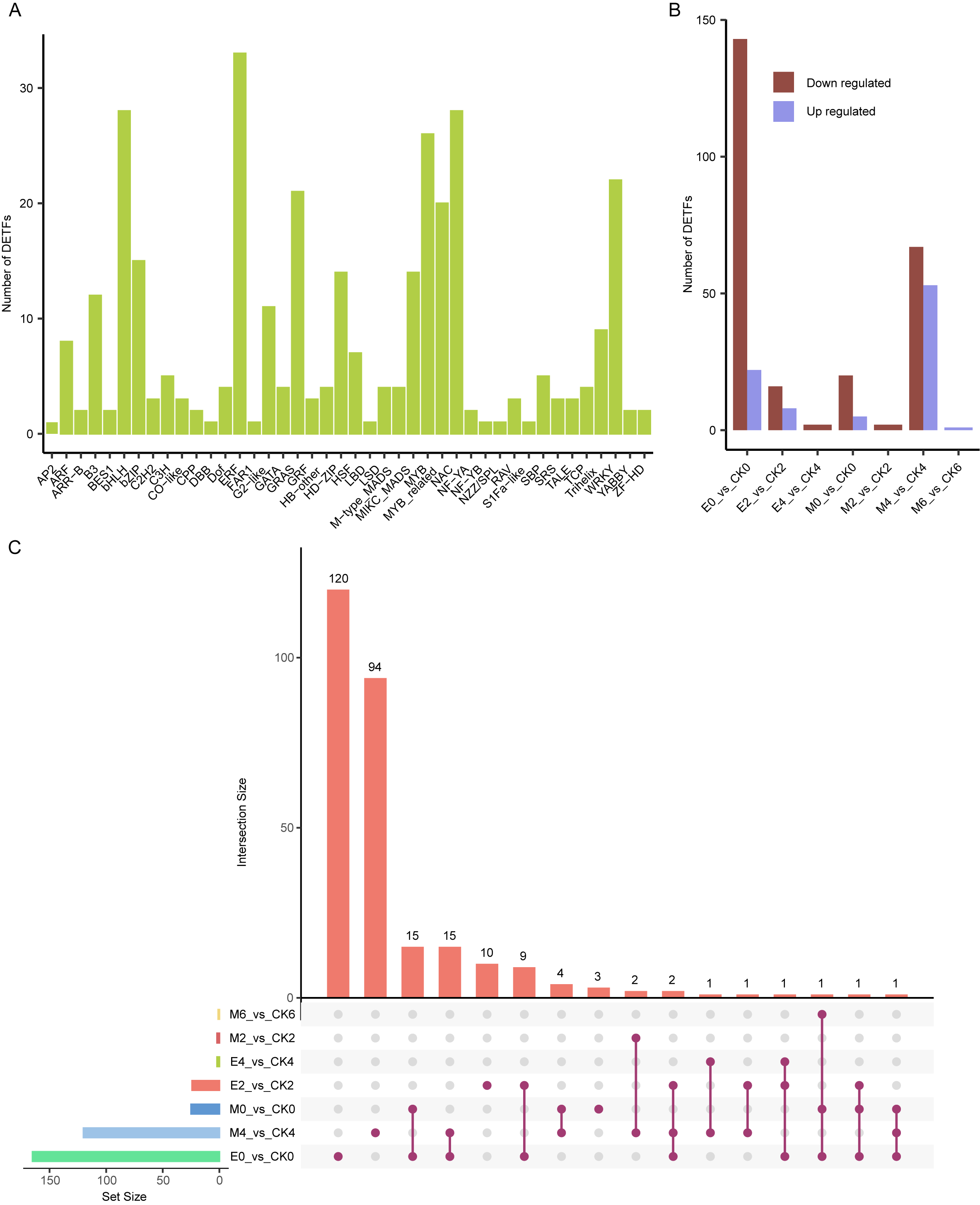
**

Supplementary Figure 1. Differential TF expression analysis in apricot fruits subjected to ethephon and 1-MCP treatments. (A) Distribution of differentially expressed TF family members; (B) Bar plot showing counts of upregulated and downregulated DETFs across comparison groups; (C) UpSet plot displaying overlapping DETFs among comparison groups. C0, C2, C4, and C6 represent the control group samples at 2, 4, 6, and 8 days of storage, respectively; E0, E2, and E4 represent the ethephon-treated group samples at 2, 4, and 6 days of storage, respectively; and M0, M2, M4, and M6 represent the 1-MCP-treated group samples at 2, 4, 6, and 8 days of storage, respectively.

**
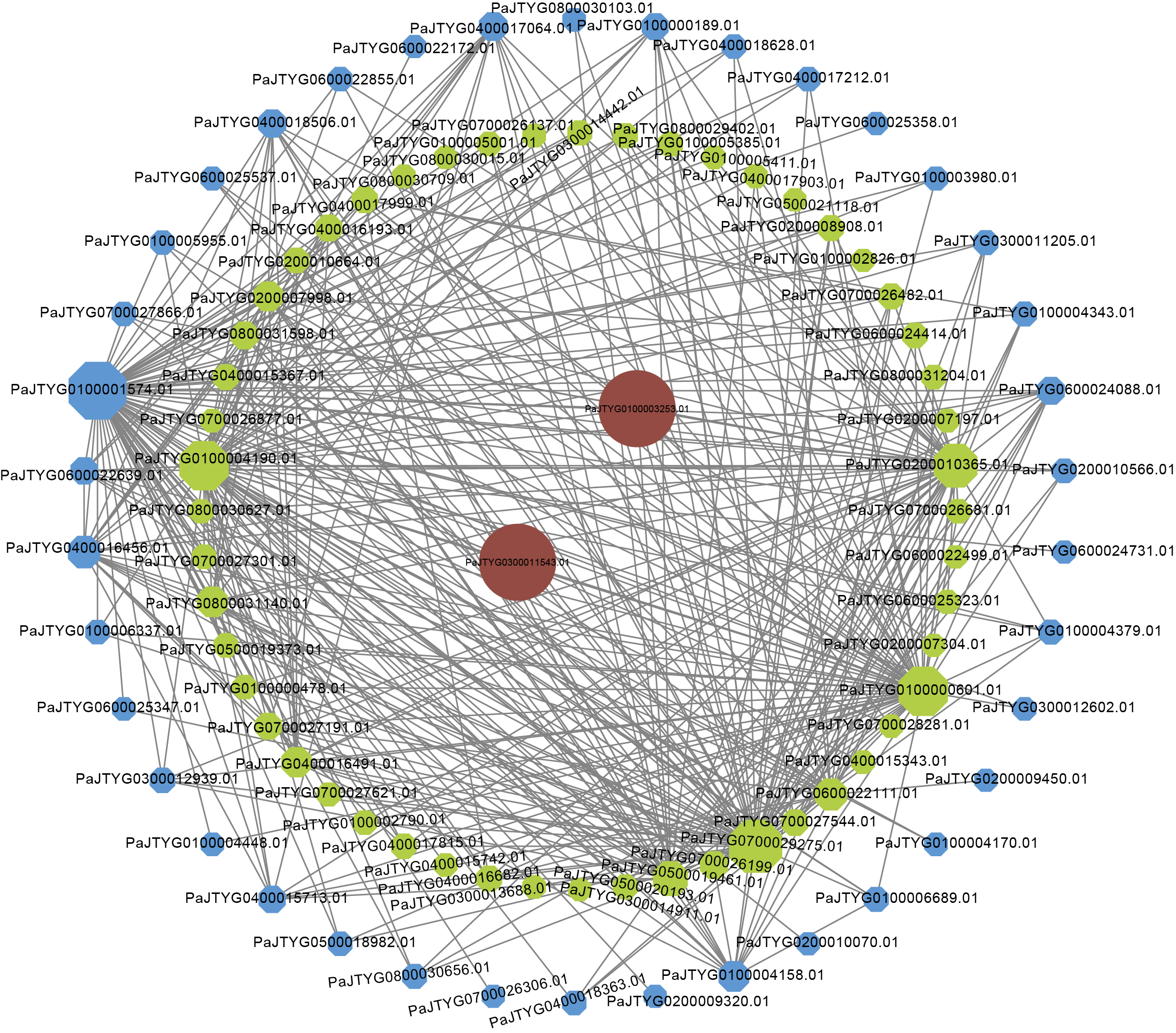
**

Supplementary Figure 2. **Gene regulatory network of the WGCNA red module.** The blue blocks represent structural genes, the green blocks denote TFs, and the red blocks indicate ethylene pathway-related genes.


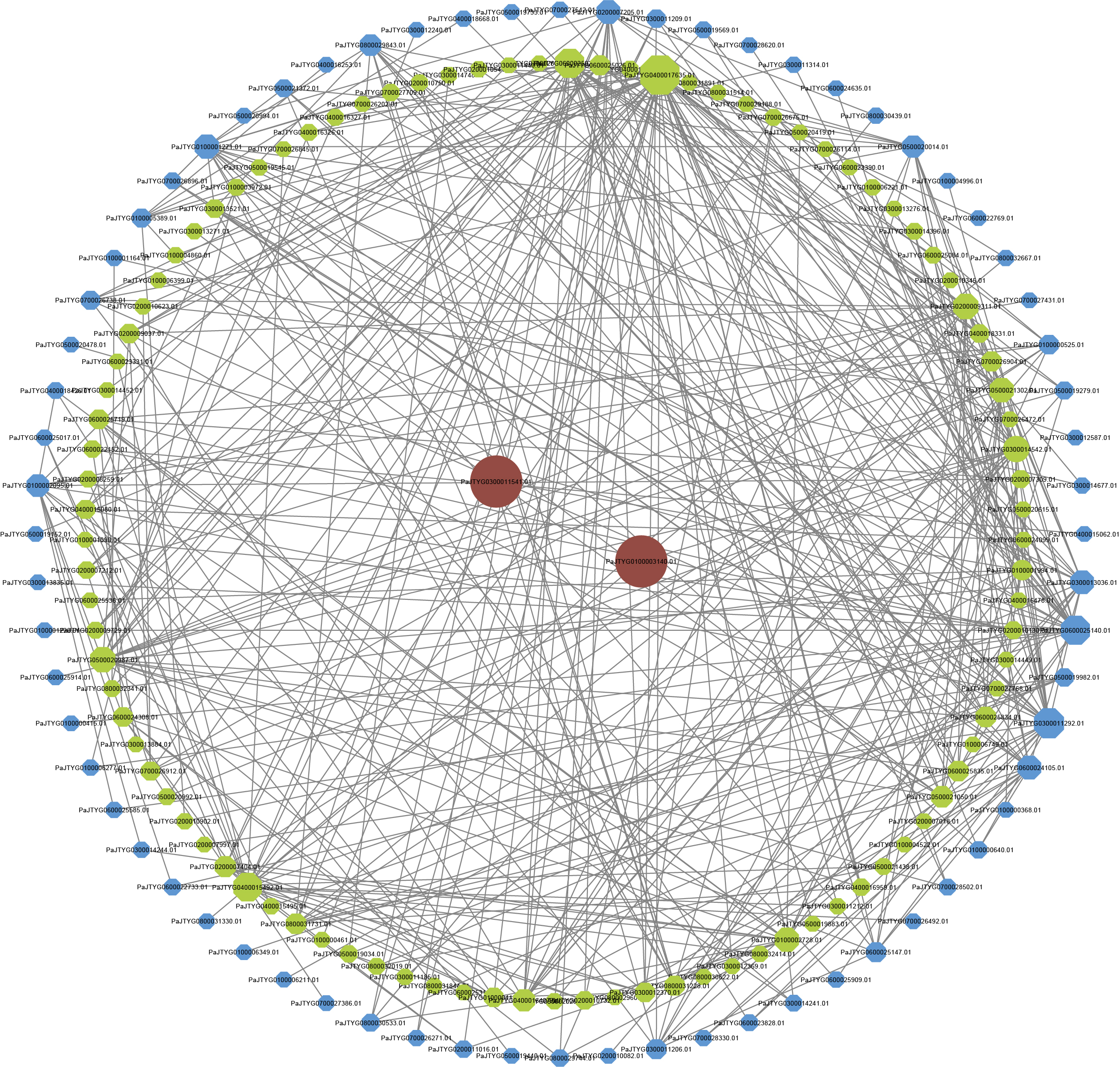


Supplementary Figure 3. **Gene regulatory network of the yellow module** identified by WGCNA. The blue blocks represent structural genes, the green blocks denote TFs, and the red blocks indicate ethylene pathway-related genes.


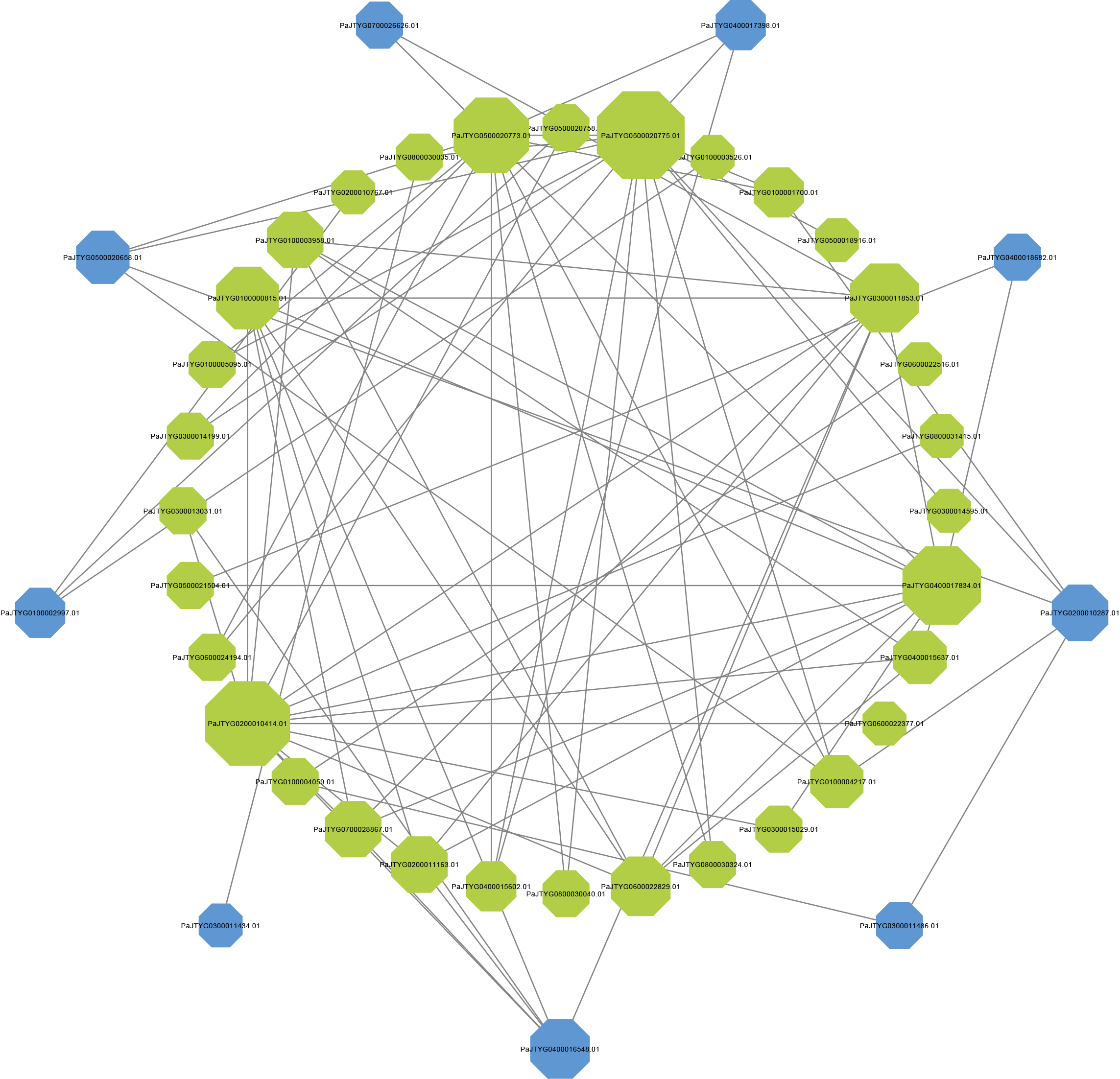


Supplementary Figure 4. **Gene regulatory network of the magenta module** identified by WGCNA. The blue blocks represent structural genes, the green blocks denote TFs, and the red blocks indicate ethylene pathway-related genes.


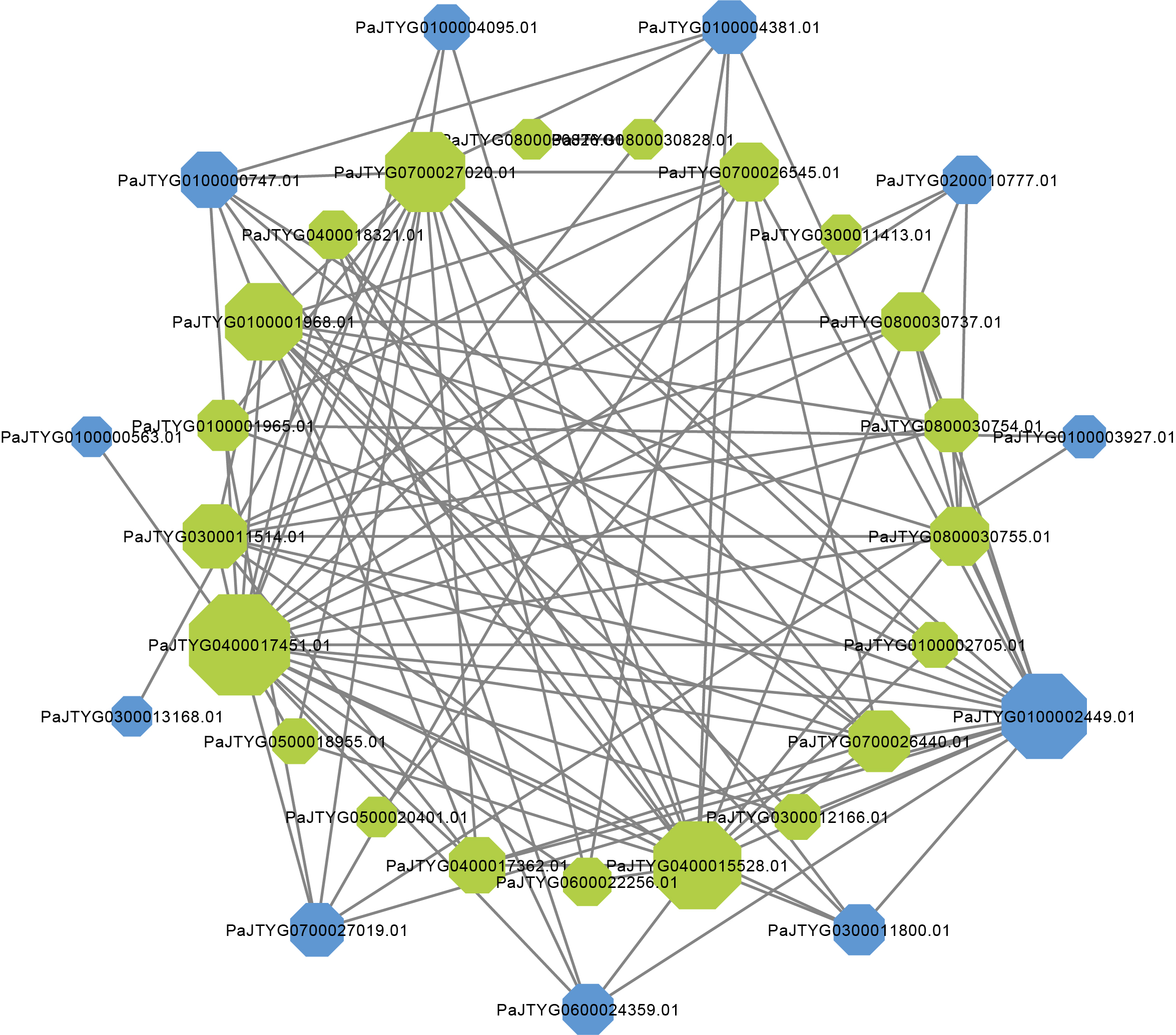


Supplementary Figure 5. **Gene regulatory network of the WGCNA tan module.** The blue blocks represent structural genes, the green blocks denote TFs, and the red blocks indicate ethylene pathway-related genes.


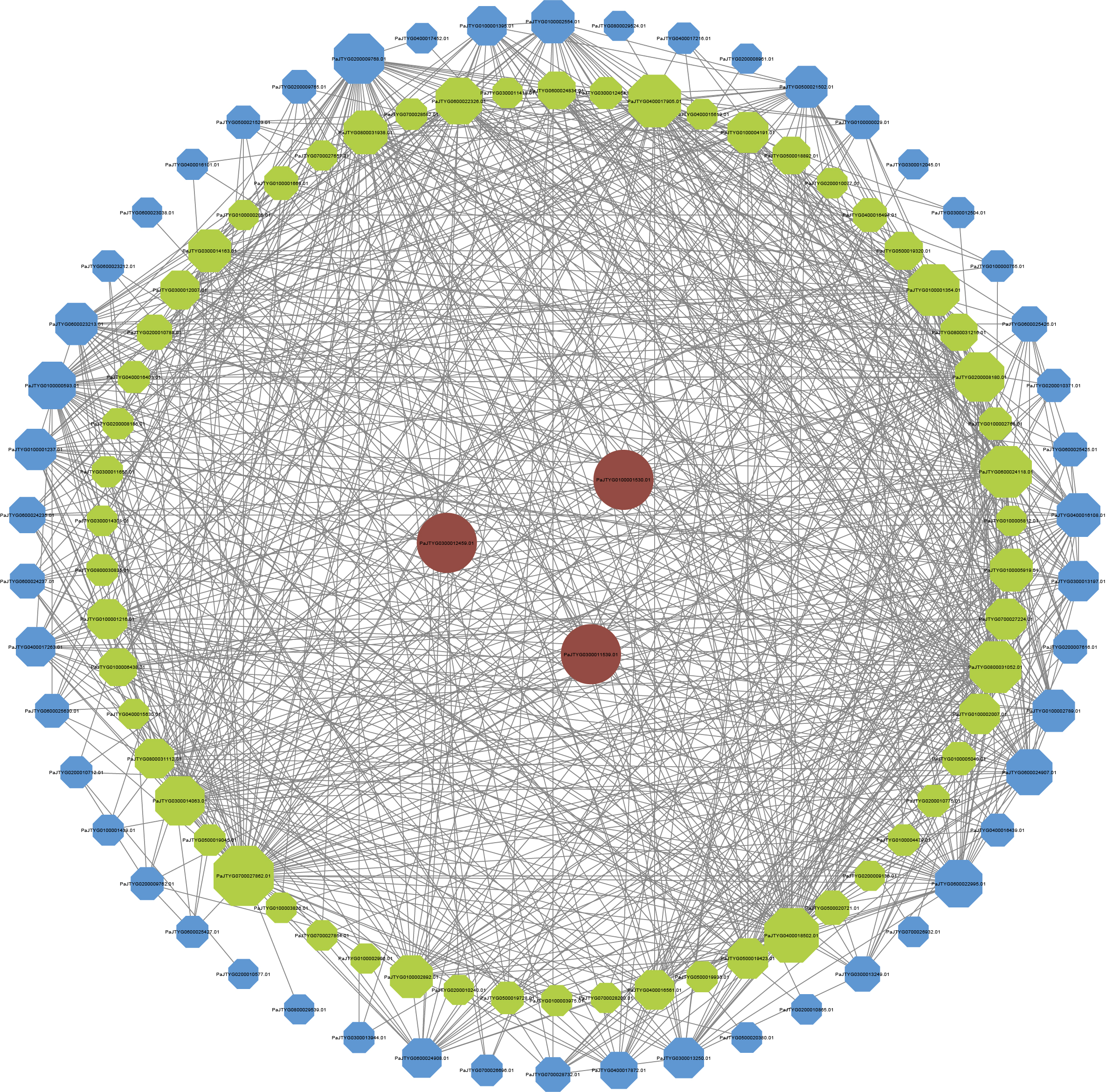


Supplementary Figure 6. **Gene regulatory network of the WGCNA green module.** The blue blocks represent structural genes, the green blocks denote TFs, and the red blocks indicate ethylene pathway-related genes.


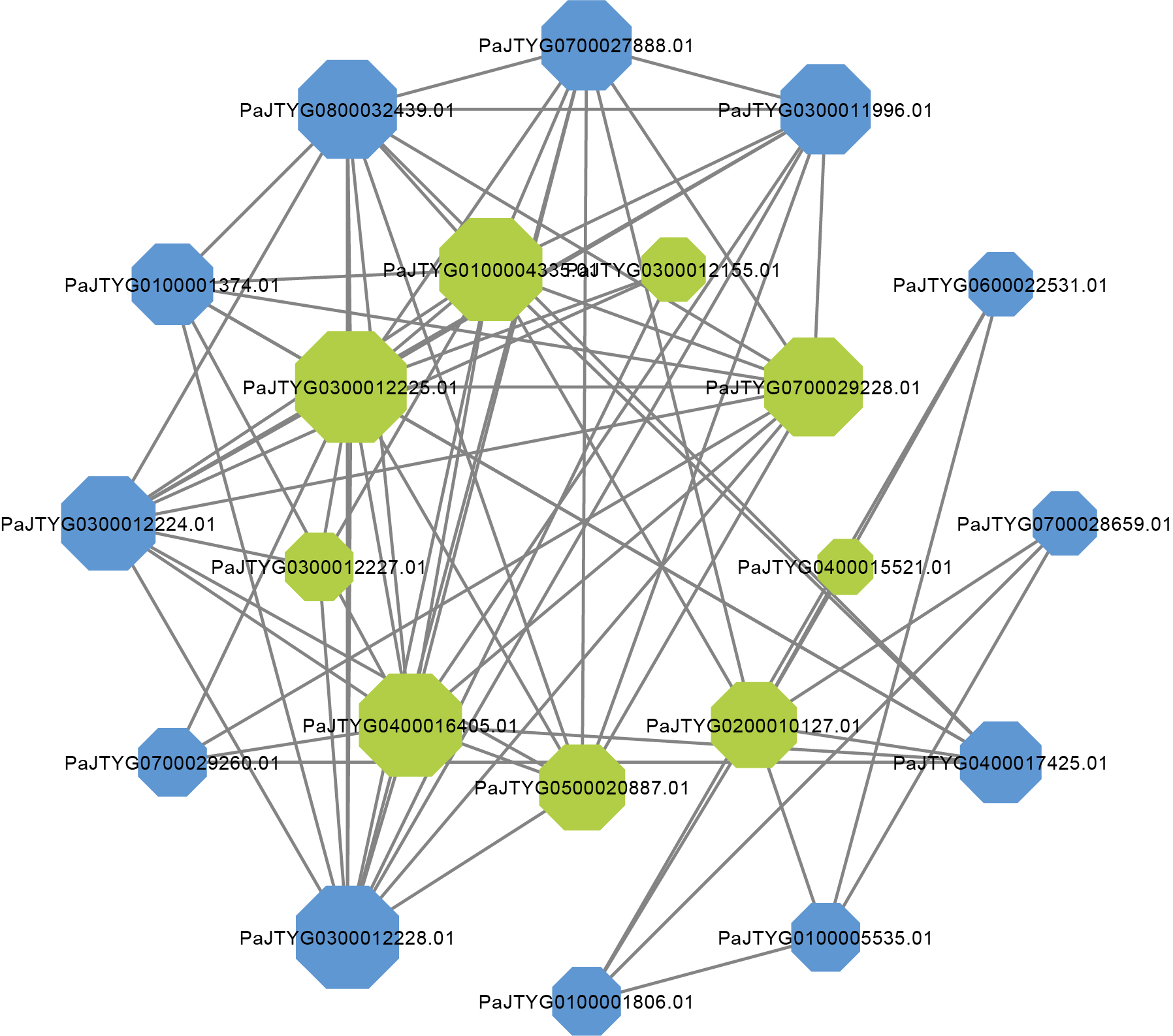


Supplementary Figure 7. **Gene regulatory network of the WGCNA salmon module.** The blue blocks represent structural genes, the green blocks denote TFs, and the red blocks indicate ethylene pathway-related genes.


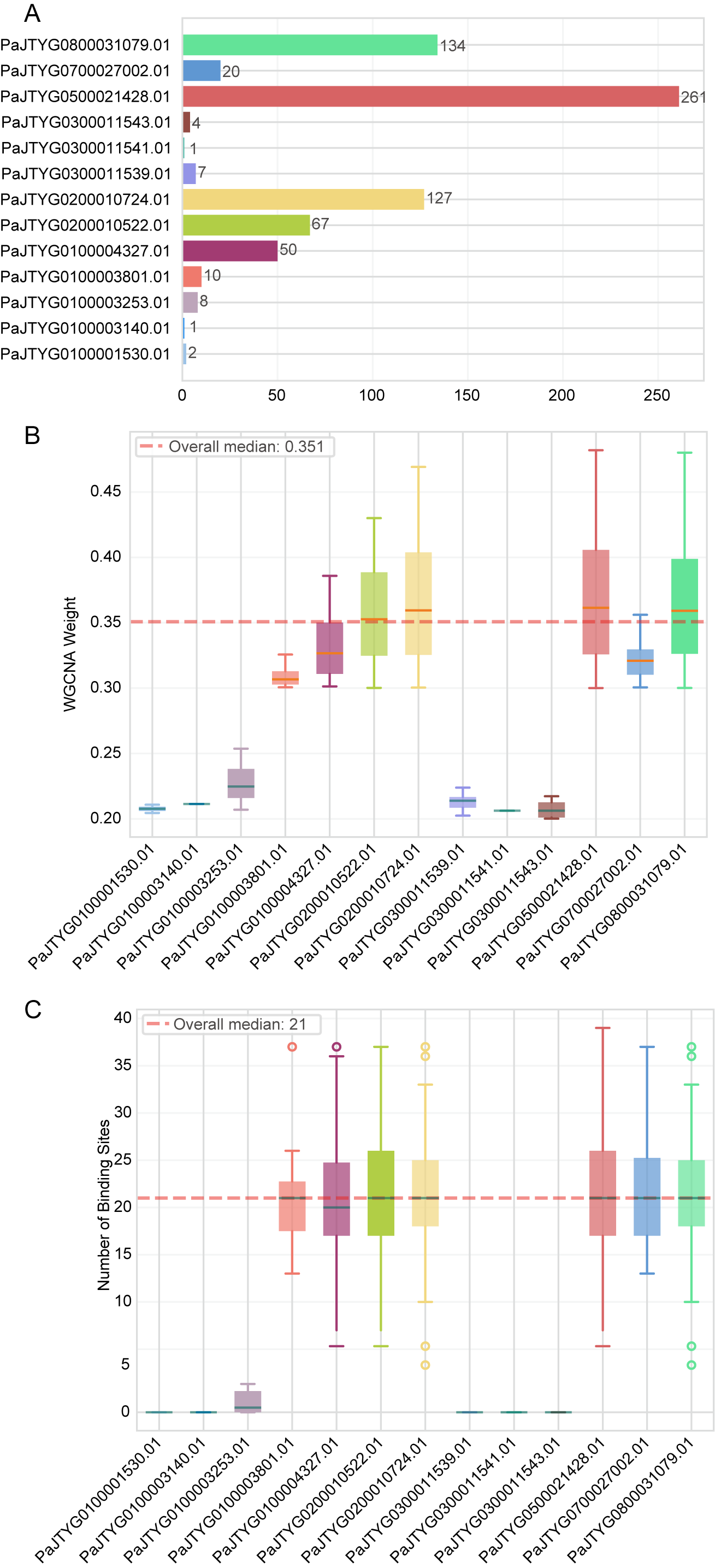


Supplementary Figure 8. **Identification of downstream target genes for eight ethylene pathway-related transcription factors.** (A) The set of genes directly associated with each target transcription factor **based on WGCNA.** (B) Weight distribution for **eight ethylene pathway-related transcription factors** **based on WGCNA.** (C) Binding sites distribution for **eight ethylene pathway-related transcription factors**. The specific counts of the identified downstream target genes are provided in Supplementary Table 3.

Supplementary Table 1. Summary of sampling time points for all experiments in this study

Supplementary Table 2. RNA-seq data quality assessment of apricot fruits treated with ethephon and 1-MCP.

Supplementary Table 3. Primers used for qRT–PCR in this study.

Supplementary Table 4. Statistical analysis of the number of identified downstream target genes for the eight screened ethylene pathway-related transcription factors.
